# The trend of mortality rates following hospitals downgrading and closures due to outbreak of COVID‐19 in Fars province: A comparative cohort study

**DOI:** 10.1002/hsr2.1850

**Published:** 2024-01-30

**Authors:** Mohammad Javad Fallahi, Sarvin Seifbehzad, Mehran Fereidooni, Amirmohammad Farrokhi, Keivan Ranjbar, Reza Shahriarirad

**Affiliations:** ^1^ Thoracic and Vascular Surgery Research Center Shiraz University of Medical Sciences Shiraz Iran; ^2^ Department of Internal Medicine Shiraz University of Medical Sciences Shiraz Iran; ^3^ Student Research Committee Shiraz University of Medical Sciences Shiraz Iran; ^4^ Legal Medicine Research Center Legal Medicine Organization Tehran Iran; ^5^ Trauma Research Center, Shahid Rajaee (Emtiaz) Trauma Hospital Shiraz University of Medical Sciences Shiraz Iran

**Keywords:** COVID‐19, hospital, mortality, pandemic, policy

## Abstract

**Background and Aims:**

Hospitals are one of the most important healthcare centers for providing the patients with different medical needs. Several different factors might cause hospitals to downgrade their services or departments or close down overall. One of the most multifaceted reasons for hospital downgrading or closure is infectious disease outbreaks. In this regard, we aimed to evaluate the effects of hospital closure and downgrading due to the COVID‐19 pandemic on the mortality rate of the people residing in Fars province, Iran.

**Methods:**

We gathered mortality information, including the cause of death, age, sex, place, and time of death of all deceased cases occurring during a period of 3 years, from February 20, 2018 to March 2021 from the forensic medicine and also the Department of Biostatistics in Shiraz University of Medical Sciences.

**Results:**

A total of 71,331 deaths have been reported since 2018 through the first quarter of 2021, with 57.9% of total mortality cases attributed to male gender. The total mortality counts ranged from 4229 to 9809 deaths per quarter, from which the minimum rate was reported in the first quarter of 2018 and the maximum in the fourth quarter of 2020. Based on the causes of death, diseases of the circulatory system were shown to be the all‐time most frequent cause of death, accounting for a total of 42.8% of recorded deaths, followed by neoplasms (9.77%) and diseases of the respiratory system (9.45%).

**Conclusion:**

Although the large number of deaths at the time of the pandemic are immediately due to COVID‐19 infection, deaths due to a notable number of other causes have had a significant increase which, along with the specific trend of place and causes of death, shows that the downgrading and closure of hospitals have had a significant impact on overall population mortality.

## Key points

- Several different causes with various natures might cause hospitals to downgrade their services or departments or close down overall, including the COVID‐19 pandemic.
- Although the large number of deaths at the time of the pandemic are immediately due to COVID‐19 infection, deaths due to a notable number of other causes have had a significant increase which, along with the specific trend of place and causes of death, shows that the downgrading and closure of hospitals have had a significant impact on overall population mortality.
- Evaluation of the long‐term impact of hospital closure on disease‐related mortalities should be continuously assessed, especially after the reopening of hospitals, to evaluate the significance of the impact of these imposed conditions.

## 1 INTRODUCTION

For centuries, healthcare policies and triage have been the backbone for medical treatment throughout the world; however, several events and situations of different natures may cause the hospitals to downgrade their activity, such as micropolitics, strikes, staff shortages, financial issues, excessive medical errors, or infectious disease outbreaks.^1^, ^2^, ^3^, ^4^, ^5^, ^6^ From 2010 to 2015, there was an average of 21 hospital closures every year in the United States, with 47 closures in 2019 alone.^7^ These changes may only be limited to emergency care and emergent surgeries, or even hospitals close down altogether. These situations vary in significance and, therefore, the impact they have on the medical services and even the future of the hospital in terms of magnitude, nature, and the duration in which they take place.

In February 2020, as the outbreak of COVID‐19 accelerated with uninterrupted transmission occurring in capital cities, the national government of Iran declared a state of emergency. They imposed strict measures designed to control the spread of the disease including mandatory hospitalization of suspected COVID‐19 cases, closure of schools and markets, restriction of public gatherings, enforcement of “safe and hygienic burial,” and internal travel restrictions and border closures. Due to the COVID‐19 pandemic financial strain on hospitals, the trend of closures accelerated, and it was anticipated that even more hospitals may close soon Association.^8^ Policymakers should thoroughly assess the repercussions and intelligently allocate budget to hospitals to lessen the negative effects of hospital closures.

The outbreak of COVID‐19 would demand peak performance from medical professionals, especially in hospitals; however, many of the facilities that may be on the front lines were closed to prevent the spread of infection. On the other hand, many patients avoided referring to hospitals due to the risk of infection; this resulted in a significant decrease in hospital admissions. These newly faced situations called for an appropriate response and alterations in healthcare management.^9^ Patients' access to proper care could significantly be disturbed by hospital closures and this condition led to increased mortality rates due to delays in receiving treatment in many studies.^10^, ^11^ Several studies revealed that hospital closures were associated with a remarkable increase in deaths, especially from trauma and heart attacks.^12^, ^13^, ^14^, ^15^ However, there are studies that found no association between hospital closures and mortality.^16^

During COVID‐19 outbreak and hospital restrictions, a proper understanding of the impact of such condition is critical as they are likely to affect the delivery of health services. Therefore, we conducted this study to evaluate the impact of Fars province hospital closures on the mortality rate in southwestern Iran.

## 2 METHODS AND MATERIALS

### 2.1 Study design

This comparative cohort study aimed to detect the impact of closures and downgrading of Fars province hospitals on the mortality rate, which occurred due to concerns regarding the COVID‐19 outbreak. The study setting of our study was Fars province, which is the fourth largest province in Iran with a population of 4,851,274 individuals (in 2016), and Shiraz, the capital of this province, has a population of 1,869,001 (the fifth most populated city in Iran). We obtained the mortality information, using data from the forensic medicine organization and the department of biostatistics from four major affiliated institutions in Fars province, consisting of Shiraz, Jahrom, Fasa, and Gerash Universities of Medical Sciences. Data were collected from February 20, 2020, to March 2021, which roughly consisted of the first year of the COVID‐19 pandemic in Fars province, along with similar data in the mentioned timeline but from 2018 to 2020 to be used for comparison. The inclusion criteria were all submitted death reports in Fars province, southern Iran, and the exclusion criteria included transferred cases from other provinces for further investigations. The information and death certificate of each case were reviewed, and their demographic features were obtained from their files including the cause of death, age, sex, date, and place of death.

### 2.2 Data collection and curation

Causes of death were gathered based on International Classification of Diseases, 10th Revision, (ICD‐10) codes and then recategorized using the ICD‐10 chapter titles, with an additional focus on COVID‐19 infection (Table 1). Causes of death were reported in sequels of four, from the initial cause to the ultimate one. The initial cause of death was gathered and labeled as the “Beginning Cause of Death (BCOD)” since it starts the causal chain of mortality, and the ultimate cause of death was labeled “Immediate Cause of Death (ICOD).” The gathered data were reported in two methods: quarterly deaths reported and binary categories before and after the COVID‐19 pandemic. To determine which causes of death were rather significant in starting the circumstances leading to death, we divided total count of the BCOD by immediate ones of the same cause. This ratio shows whether a cause of death is more directly responsible for mortality if its value is less than 1 and vice versa if it is more than 1. The age of our cases were categorized into seven groups, including infants (up to 1 year old), children (1−12 years old), teens (13−18 years old), young adults (19−25 years old), middle‐aged adults (26−45 years old), senior adults (46−65 years old), and old age (over 65 years old). The place of death of our cases was categorized into two main groups, including inpatient deaths (death in a healthcare center or hospital) and outpatient deaths (deaths occurring in places other than healthcare centers).

**Table 1:** Categorization of diseases based on ICD10‐code.

#	Chapter	ICD‐10 code
0	Missing	‐
1	Certain infectious and parasitic diseases	A00‐B99
2	Neoplasms	C00‐D49
3	Diseases of the blood and blood‐forming organs and certain disorders involving the immune mechanism	D50‐D89
4	Endocrine, nutritional, and metabolic diseases	E00‐E89
5	Mental, behavioral, and neurodevelopmental disorders	F01‐F99
6	Diseases of the nervous system	G00‐G99
7	Diseases of the eye and adnexa	H00‐H59
8	Diseases of the ear and mastoid process	H60‐H95
9	Diseases of the circulatory system	I100‐I99
10	Diseases of the respiratory system	J00‐J99
11	Diseases of the digestive system	K00‐K95
12	Diseases of the skin and subcutaneous tissue	L00‐L99
13	Diseases of the musculoskeletal system and connective tissue	M00‐M99
14	Diseases of the genitourinary system	N00‐N99
15	Pregnancy, childbirth, and the puerperium	O00‐O9A
16	Certain conditions originating in the perinatal period	P00‐P96
17	Congenital malformations, deformations, and chromosomal abnormalities	Q00‐Q99
18	Symptoms, signs, and abnormal clinical and laboratory findings, not elsewhere classified	R00‐R99
19	Injury, poisoning, and certain other consequences of external causes	S00‐T88
20	Codes for special purposes	U00‐U85
21	External causes of morbidity (i.e., motor vehicle accidents, assault, and self‐harm)	V00‐Y99
22	Factors influencing health status and contact with health services	Z00‐Z99
23	COVID‐19	U07.1‐U07.2

### 2.3 Data analyses

The collected data were entered into Excel 2021 (Microsoft Software) and SPSS version 26.0 (IBM Software) software and evaluated for normality using the Kolmogorov−Smirnov normality test. After data cleansing, we analyzed and reported the results, using frequency and percentage distribution diagrams for descriptive indicators and mean and standard deviation for numerical values.

## 3 RESULTS

### 3.1 Overall mortality trends and features

**Figure 1:**
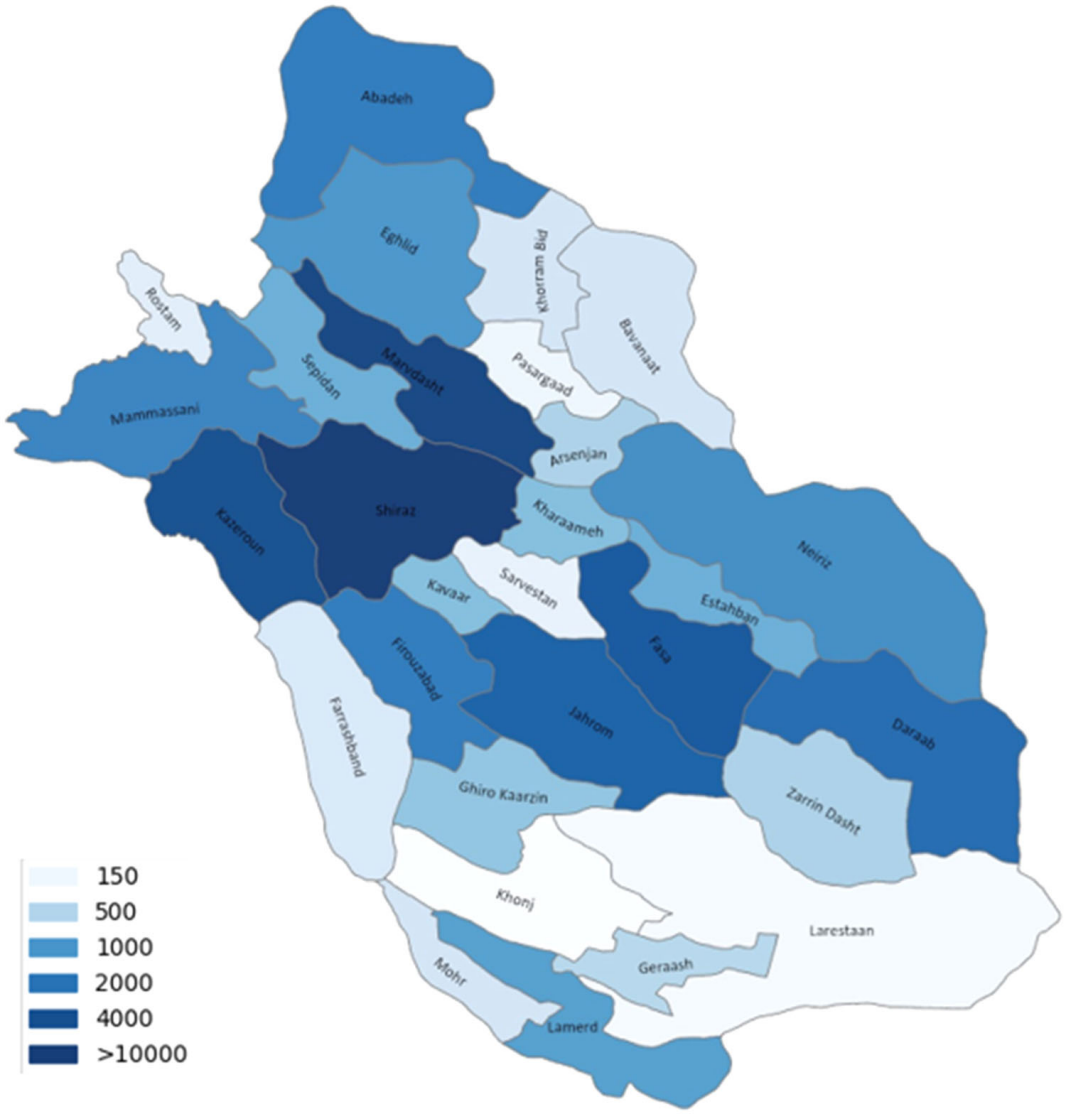
Geographic information map based on the total death counts by cities in Fars province from February 2018 till March 2021 (Shiraz is the capital of the province).

**Figure 2:**
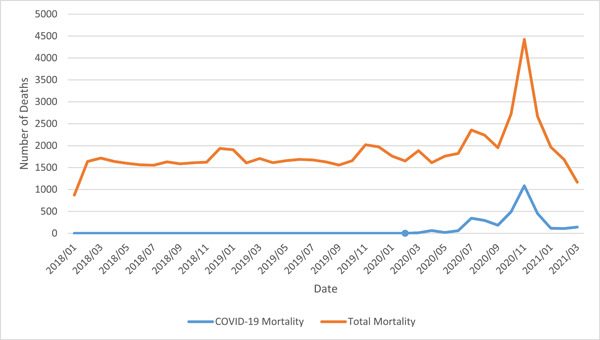
Frequency of mortality in Fars province, Southwestern Iran (*indicator of the start of the COVID‐19 pandemic).

A total of 67,867 deaths were reported in Fars province, Iran, from 2018 to the first quarter of 2021, with 57.8% of total mortalities attributed to male gender. Mortalities reported in this time span had an upper age‐border of 105 with an average of 66.6 ± 22.7 years. Total mortality counts ranged from 2164 to 9809 deaths per quarter, from which the minimum was reported in the first quarter of 2020 and the maximum in the fourth quarter of 2020 (Table 2). The mortality rate in the cities in our province, based on the geographic information map, are demonstrated in Figure 1, while the trend of mortalities throughout the period of our study is displayed in Figure 2.

**Table 2:** Frequency and changes of mortality based on timeline and quarterly (Q), half‐year, and full‐year categorization (*N* = 70,980).

Year	Total, *n* (%)	Half year		Quarterly			
		First	Second	Q1	Q2	Q3	Q4
2018	18,970 (26.59)	9029 (12.66)	9941 (13.94)	4229 (5.93)	4800 (6.73)	4771 (6.69)	5170 (7.25)
2019	20,686 (29)	10,174 (14.26)	10,512 (14.74)	5220 (7.32)	4954 (6.95)	4863 (6.82)	5649 (7.92)
2020	26,858 (37.65)	10,494 (14.71)	16,364 (22.94)	5300 (7.43)	5194 (7.28)	6548 (9.18)	9816 (13.76)
2021	‐	‐	‐	4817 (6.75)	‐	‐	‐

The gender distribution among the two genders remained roughly unchanged with an average of 57.9% of deaths attributed to males, and a quarterly range of 56.2%−59.5%. Comparison of the impact of gender based on male‐to‐female ratio showed that in the category codes “Endocrine, nutritional, and metabolic disorders,” “Congenital malformations, deformations, and chromosomal abnormalities,” “Diseases of the skin and subcutaneous tissue,” “Certain conditions originating in the perinatal period,” and “Diseases of the musculoskeletal system and connective tissue” had a more impactful role in female mortality rates (Table 3).

**Table 3:** Distribution of mortality based on the cause and gender.

ICD code	Code	Frequency (%)	Male/female (ratio)	BCOD	BCOD/ICOD
9	Diseases of the circulatory system	30,531 (42.80)	16,437/14,094 (1.17)	27,841	0.91
2	Neoplasms	6969 (9.77)	3998/2971 (1.35)	8772	1.26
10	Diseases of the respiratory system	6739 (9.45)	3956/2783 (1.42)	4594	0.68
21	External causes of morbidity	6434 (9.02)	5086/1348 (3.77)	7572	1.18
18	Symptoms, signs, and abnormal clinical and laboratory findings, not elsewhere classified	4057 (5.69)	2566/1491 (1.72)	4268	1.05
1	Certain infectious and parasitic diseases	3570 (5.00)	1933/1637 (1.18)	1289	0.36
23	COVID‐19 infection	3203 (4.49)	1892/1311 (1.44)	5040	1.57
0	Missing	2106 (2.95)	1183/923 (1.28)	2106	1.00
14	Diseases of the genitourinary system	1754 (2.46)	926/828 (1.12)	1753	1.00
11	Diseases of the digestive system	1604 (2.25)	969/635 (1.53)	1899	1.18
6	Diseases of the nervous system	1129 (1.58)	602/527 (1.14)	1503	1.33
4	Endocrine, nutritional, and metabolic diseases	965 (1.35)	446/519 (0.86)	2280	2.36
19	Injury, poisoning, and certain other consequences of external causes	576 (0.81)	448/128 (3.50)	206	0.36
5	Mental, behavioral, and neurodevelopmental disorders	470 (0.66)	339/131 (2.59)	639	1.36
17	Congenital malformations, deformations, and chromosomal abnormalities	376 (0.53)	176/200 (0.88)	469	1.25
16	Certain conditions originating in the perinatal period	328 (0.46)	124/204 (0.61)	342	1.04
3	Diseases of the blood and blood‐forming organs and certain disorders involving the immune mechanism	270 (0.38)	142/128 (1.11)	248	0.92
12	Diseases of the skin and subcutaneous tissue	145 (0.20)	66/79 (0.84)	207	1.43
13	Diseases of the musculoskeletal system and connective tissue	72 (0.10)	22/50 (0.44)	233	3.24
15	Pregnancy, childbirth, and the puerperium	25 (0.04)	0/25 (0.00)	33	1.32
22	Factors influencing health status and contact with health services	6 (0.01)	5/1 (5.0)	35	5.83
8	Diseases of the ear and mastoid process	1 (0.00)	1/0 (0)	2	2.00
20	Codes for special purposes	1 (0.00)	0/1 (0)	0	0.00
7	Diseases of the eye and adnexa	0 (0.00)	0/0 (0)	0	N/A

*Note*: Cause of death are described as the beginning cause of death (BCOD) since it starts the causal chain of mortality, and the ultimate cause of death is labeled “immediate cause of death (ICOD).”

Abbreviation: ICD‐10, International Classification of Diseases 10th Revision.

The BCOD/ICOD ratio based on our data is reported in Table 3; as demonstrated, diseases of the circulatory system and the respiratory system showed a ratio of 0.91 and 0.63 respectively, indicating that these causes of death were more an ultimate cause. COVID‐19 and neoplasms had a ratio greater than 1, implying that these causes rather began the circumstance that led to death.

### 3.2 Time trend of causes of mortality

**Figure 3:**
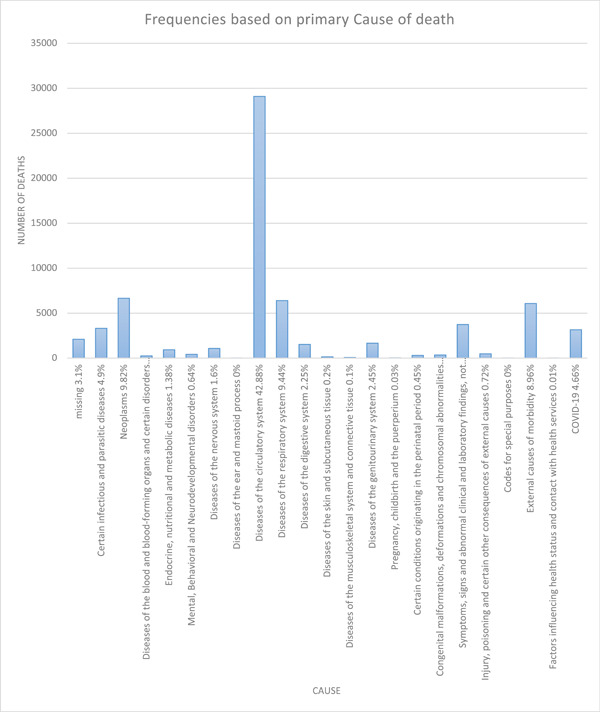
Frequency of causes of death in Fars province, Southwestern Iran from February 2018 till March 2021.

**Figure 4:**
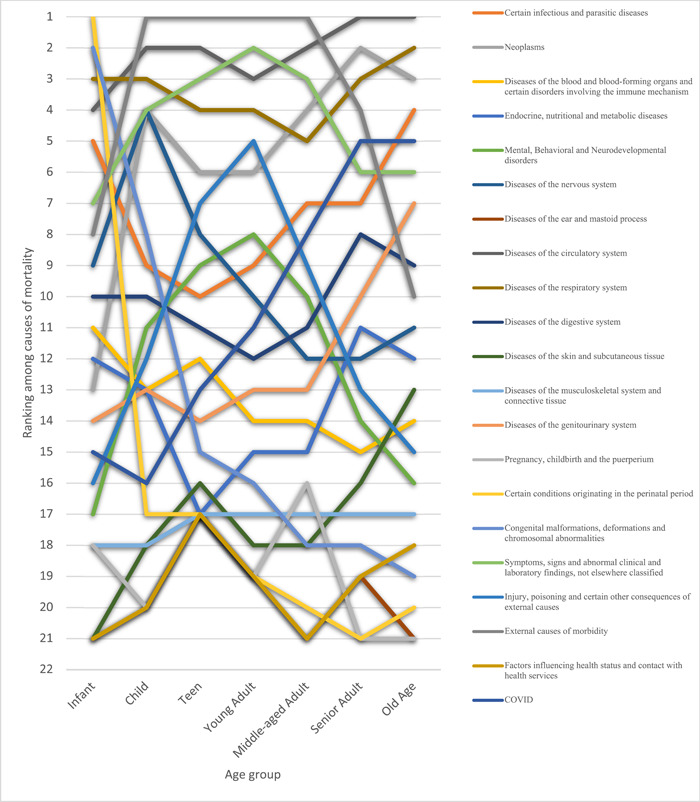
Ranking of the cause of death among various age groups in Fars, Iran from February 2018 to March 2021.

Based on the causes of death, diseases of the circulatory system were the all‐time most frequent cause, accountable for a total of 42.9% of deaths recorded, followed by neoplasms (9.82%) and diseases of the respiratory system (9.44%). The rank of diseases of the circulatory system was unchanged through all quarters in this study. Other causes, however, showed a variation, with neoplasms being the second overall but changing to being the third due to external causes of morbidity in the third quarter of 2018; also, after the first quarter of 2020, they were replaced by external causes of mortality, COVID‐19 infection, and respiratory diseases. The third overall ICOD was diseases of the respiratory system, which had become more prominent since the first quarter of 2020. External causes of morbidity which included motor vehicle accidents, assault, and self‐harm were the fourth most frequently reported ICOD, with a drop in frequency in the third and fourth quarters of 2020. Injury, poisoning, and certain other consequences of other causes showed a slight rise in rate in the first quarter of 2020. Deaths due to pregnancy and childbirth had a total number of 22 in the course of this study, with a maximum of six deaths in the first quarter of 2019. The overall causes of mortality are demonstrated in Figure 3, and their ranking throughout the period of our study is illustrated in Figure 4.

### 3.3 Mortality trend based on age groups

The mortality rate increased gradually with age, from 1.6% of deaths in the infant category to 61.1% in old age (Table 4). Observations across age categories and causes of death showed worrying facts, especially in the teen and young adult age category. In the infant age category, the main causes of death were the conditions originating in the perinatal period and congenital malformations as expected, representing 51.4% of total deaths in the infancy period. Diseases of the respiratory and circulatory systems follow afterward, being accountable for 8.9% and 7.4% of infantile deaths, respectively.

**Table 4:** Distribution and changes of mortality based on quartile among age groups.

Age group (years)	Total (%)	2018				2019				2020				2021
		Q1	Q2	Q3	Q4	Q1	Q2	Q3	Q4	Q1	Q2	Q3	Q4	Q1
Infant (<1)	1146 (1.61)	85	112	77	97	98	107	108	111	102	53	77	74	45
Change (%)			31.76	−31.25	25.97	1.03	9.18	0.93	2.78	−8.11	−48.04	45.28	−3.90	−39.19
Child (1–12)	1227 (1.72)	87	108	94	103	89	100	114	113	70	88	103	97	61
Change (%)			24.14	−12.96	9.57	−13.59	12.36	14.00	−0.88	−38.05	25.71	17.05	−5.83	−37.11
Teen (13–18)	919 (1.29)	68	60	65	47	60	71	70	74	73	104	91	69	67
Change (%)			−11.76	8.33	−27.69	27.66	18.33	−1.41	5.71	−1.35	42.47	−12.50	−24.18	−2.90
Young adult (19–25)	1576 (2.21)	103	157	144	113	110	87	117	117	166	116	132	134	80
Change (%)			52.43	−8.28	−21.53	−2.65	−20.91	34.48	0.00	41.88	−30.12	13.79	1.52	−40.30
Middle‐aged adult (26 – 45)	7460 (10.46)	447	566	624	575	508	544	525	540	569	594	687	779	502
Change (%)			26.62	10.25	−7.85	−11.65	7.09	−3.49	2.86	5.37	4.39	15.66	13.39	−35.56
Senior adult (46–65)	15,251 (21.38)	857	1024	999	1103	1127	1034	954	1214	1060	1127	1493	2203	1056
Change (%)			19.49	−2.44	10.41	2.18	−8.25	−7.74	27.25	−12.69	6.32	32.48	47.56	−52.07
Old age (>65)	43,752 (61.34)	2582	2773	2768	3132	3228	3011	2975	3480	3260	3112	3965	6460	3006
Change (%)			7.40	−0.18	13.15	3.07	−6.72	−1.20	16.97	−6.32	−4.54	27.41	62.93	−53.47

In the child age category, external causes of mortality followed by diseases of the circulatory and respiratory systems were the top three causes of death with 31.7%, 10.4%, and 8.3% of total deaths. Neoplasms follow these three causes and continue to be a rather prominent cause of death in all age categories except infants. Diseases of the nervous system and “symptoms, signs, and abnormal clinical and laboratory findings, not elsewhere classified” are as prevalent as neoplasms in the child age group, each accounting for 6.2% of total deaths.

In the teenage category, a similar pattern with younger age groups was observed, with almost the same causes being responsible for the highest number of deaths. External causes of mortality constituted a rate of 53% of all deaths reported, while diseases of the circulatory system comprised 9.7%. “Symptoms, signs, and abnormal clinical and laboratory findings, not elsewhere classified” were in the third rank, with a rate of 7.6%. A notable fact is that the teenage category constituted the least number of mortalities reported amongst age categories, with 872 deaths out of a total of 67,867 (1.2%).

External causes of mortality remained to be the first cause of death in the young adult age range with a high margin (56.8%), followed by “symptoms, signs, and abnormal clinical and laboratory findings, not elsewhere classified” (11%) and diseases of the circulatory system (10.7%). Diseases of the circulatory system ranked second to external causes of mortality in middle‐aged adults. From this age range, a gradual rise was observed in death counts due to diseases of the circulatory system, contributing to 16.5% of deaths. External causes of mortality, however, made up 33.2% of deaths, still being the first. “Symptoms, signs, and abnormal clinical and laboratory findings, not elsewhere classified” came third with almost the same rate as younger age categories (11.4%), and death due to neoplasms started to become considerably more since this age (9%). All deaths due to pregnancy and childbirth occurred in middle‐aged women. Senior adults and people of old age contributed the most to the overall ranking of causes of death, in such a manner that the mortality rate exceeded the total sum of all younger age groups (14,627 and 41,497 vs. 11,743 deaths, respectively). Diseases of the circulatory system was the leading cause of death with a rate of 36.8% and 53.2% in senior adults and old age adults, respectively. Neoplasms was the second, with the highest rank in all age categories and 16.2% of deaths in senior adults. Diseases of the respiratory system were in the third rank, with 9.1% of deaths. Following closely was external causes of mortality with a rate of 8.6%. The second rank amongst the old age population belonged to the diseases of the respiratory system, with a rate of 10.5%, which illustrates a wide gap of 17,726 death counts (42.7%) with the previous cause. The third cause of death was neoplasms which caused the highest number of deaths compared to other age categories (3490 deaths), still having a lesser rate of 8.4%. Deaths due to external causes of mortality showed a sharp drop to only 735% or 1.7%. However, infectious, and parasitic diseases were the fourth most common cause of death in people older than 65 years old. There was also a notable 98% decrease in the cause of death labeled as missing throughout this time (Figure 4).

### 3.4 Impact of COVID‐19 on mortality

**Figure 5:**
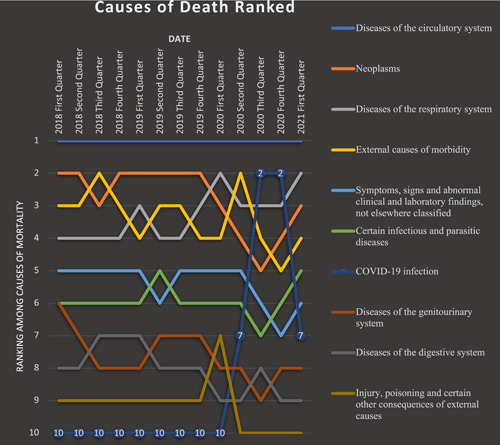
Ranking of causes of death from 2018 to 2021 in Fars province, Iran.

Since its first report in February 2020, COVID‐19 had a major role in increasing the quarterly reported mortality rates, being the second cause of death reported in the third and fourth quarters of 2020 and seventh in the early 2020 and 2021. COVID‐19 was first reported in China on December 31, 2019, while the first case in Iran was in Qom city on February 19, 2020; then, it took 12 days for the first case to be reported in Fars in Shiraz. Since then, COVID‐19 had a prominent role in mortality rates in Fars province. Being responsible for a cumulative count of 3162 deaths (4.6%), COVID‐19 has had an impact on causes of death, rapidly replacing other causes of death. To evaluate this more thoroughly, deaths were divided into two categories based on the pandemic, as mentioned in the method section, and total deaths before and after it were divided according to the number of quarters each part endured, to better show the impact COVID‐19 had on Fars province mortalities. We explored this starting with diseases of the circulatory system that had a total count of 17,247 deaths over the course of eight quarters before the COVID‐19 pandemic. This shows an average of 2155.8 deaths per quarter, where afterward the sum is 11,851 in five quarters, and the average increases to 2370.2 per quarter. The per‐quarter average of the top 10 causes of mortality and their changes before and after the COVID‐19 era is illustrated in Table 5 and Figure 5. What can be inferred from this illustration is that COVID‐19 infection and other diseases of the respiratory system had a sharp increase in quarterly average deaths and replace neoplasms and external causes of mortality. Other causes of mortality, however, only show an increase by one in rank caused by COVID‐19.

**Table 5:** Top 10 causes of death based on the COVID‐19 pandemic timeline.

ICD code	Code	Before COVID‐19					During COVID‐19					Total
		Total	Average	Rank	Inpatient	Outpatient	total	Average	Rank	Inpatient	Outpatient	
9	Diseases of the circulatory system	17,345	2168.13	1	7751	9594	13186	2637.20	1	4925	8261	30,531
2	Neoplasms	4563	570.38	2	2600	1963	2406	481.20	4	1002	1404	6969
10	Diseases of the respiratory system	3204	400.50	4	1883	1321	3535	707.00	2	2650	885	6739
21	External causes of morbidity	4040	505.00	3	1600	2440	2394	478.80	5	1035	1359	6434
18	Symptoms, signs, and abnormal clinical and laboratory findings, not elsewhere classified	2153	269.13	5	866	1287	1904	380.80	6	790	1114	4057
1	Certain infectious and parasitic diseases	1717	214.63	7	1248	469	1853	370.60	7	1302	551	3570
23	COVID‐19 infection	0	0.00	23	0	0	3203	640.60	3	2920	283	3203
0	Missing	1891	236.38	6	776	1115	215	43.00	14	99	116	2106
14	Diseases of the genitourinary system	1103	137.88	8	652	451	651	130.20	8	369	282	1754
11	Diseases of the digestive system	1048	131.00	9	775	273	556	111.20	9	364	192	1604
6	Diseases of the nervous system	656	82.00	10	294	362	473	94.60	10	170	303	1129
4	Endocrine, nutritional, and metabolic diseases	597	74.63	11	346	251	368	73.60	11	159	209	965
19	Injury, poisoning, and certain other consequences of external causes	282	35.25	12	128	154	294	58.80	12	172	122	576
5	Mental, behavioral, and neurodevelopmental disorders	254	31.75	14	82	172	216	43.20	13	80	136	470
17	Congenital malformations, deformations, and chromosomal abnormalities	266	33.25	13	188	78	110	22.00	17	81	29	376
16	Certain conditions originating in the perinatal period	217	27.13	15	188	29	111	22.20	15	88	23	328
3	Diseases of the blood and blood‐forming organs and certain disorders involving the immune mechanism	159	19.88	16	139	20	111	22.20	15	90	21	270
12	Diseases of the skin and subcutaneous tissue	88	11.00	17	34	54	57	11.40	18	10	47	145
13	Diseases of the musculoskeletal system and connective tissue	46	5.75	18	21	25	26	5.20	19	16	10	72
15	Pregnancy, childbirth, and the puerperium	19	2.38	19	14	5	6	1.20	20	3	3	25
22	Factors influencing health status and contact with health services	6	0.75	20	3	3	0	0.00	21	0	0	6
8	Diseases of the ear and mastoid process	1	0.13	21	1	0	0	0.00	21	0	0	1
20	Codes for special purposes	1	0.13	21	1	0	0	0.00	21	0	0	1
7	Diseases of the eye and adnexa	0	0.00	23	0	0	0	0.00	21	0	0	0

## 4 DISCUSSION

This study aimed to evaluate the mortality rate during hospital downgrading due to the COVID‐19 pandemic and compare it with the same previous time frames. Before the pandemic, the crude death rate had a relatively steady trend, ranging from 1601 to 1978 in each month. After the onset of the pandemic in every peak, which brought about general lockdowns and stricter regulations, monthly mortality rates had a significant increase, reaching 4421 in November 2020 during the very strict transportation regulations, which is more than twice the mortality rates before the pandemic and lockdowns.

The first COVID‐19 peak started in the middle of the first quarter of 2020 and lasted until the middle of the second quarter of 2020. The last month of the second quarter of 2020 was the second peak, and the fourth quarter of 2020 was roughly the third peak of COVID‐19 in Iran. Although after the second peak, the slope in the mortality diagram was relatively lower for a short period of time, the mortality trend continued to rise. In the third peak, mortality and its trend reached an all‐time high, so that in the middle of the fourth quarter, monthly mortality reached a rate higher than 2.7 times the mean mortality rate in the period before the pandemic.

As we demonstrated in the COVID‐19 statistics in our results, we experienced three peaks during our study period, and there was a shift from inpatient COVID‐19 to outpatient management of the disease. The statistics regarding COVID‐19 deaths are most probably under‐reported, due to a lack of reporting possibly caused by stigma, fear of compulsory quarantine for relatives, hospital expenses or neglect, and a genuine under‐reporting caused by the lack of documented evidence of COVID‐19 infection. This has also been stated by official reports and would mean that a notable proportion of COVID‐19‐related deaths have been registered as other causes, suggesting that the massive increase in non‐COVID‐19 deaths might be partly due to these reasons.

The mortality trend was on the rise during the pandemic and lockdowns; although the mortality rate had a steady trend in the 26 months before the pandemic, it ascended during the 13‐month pandemic period by 33%, compared to the mean crude death rate in previous periods. Considering the increase in non‐COVID‐19 deaths in this period, it can be argued that the lockdowns and downregulations have a negative impact on the mortality rate. Another attributing factor could be the fear of seeking medical care at the time of the pandemic.

The three main causes of death in patients before and during the pandemic are diseases of the circulatory system, neoplasms, and diseases of the respiratory system. With the onset of the pandemic, mortality increased in most causes of death. Although COVID‐19 has become the second most common cause of death in two quarters and death due to respiratory diseases has increased, the rise in mortality rate is not solely due to these two reasons. The highest death rate due to COVID‐19 was in November 2020 with 1084 deaths, making up 24.51% of the total deaths.

Deaths due to neoplasms had a drastic drop at the beginning of the pandemic, but as time went by, it returned to its previous trend. It might be possible that this temporary decrease in the number of deaths due to neoplasms is because of fewer admissions of cancer patients at the beginning of the pandemic and the delay in treatments like chemotherapy and surgical treatments.

The third overall ICOD was diseases of the respiratory system, which have become more prominent since the first quarter of 2020. This increase could be linked to cases of undiagnosed COVID‐19, especially at the beginning of the pandemic, due to the lack of accurate diagnostic tests. On the other hand, deaths related to the digestive and genitourinary systems and injury/poisoning showed a decrease at the time of the pandemic, except for the first quarter of 2020 which witnessed a rise in deaths by injury/poisoning, due to a surge in methanol poisonings.

Analyses of BCOD/ICOD show that COVID‐19, like neoplasms, is mostly the initiator of the cascade that results in death. On the other hand, diseases of the circulatory system, diseases of the respiratory system, and certain infections have ratio values less than 1, which shows they are mostly the ultimate cause of death.

Among the other published reports on the applied policies and downgrading, one case was mentioned from a private transplant referral hospital in our province.^9^ The main findings demonstrated that the COVID‐19 pandemic had a discernible impact in terms of postponing surgery and raising hospital expenses and workload. However, by implementation of proper policies, early diagnosis for COVID‐19, and application of the required treatment and preventative protocols to protect the patients, the center was able to deliver satisfactory medical services for patients and limit the spread of infection.^9^ In a report from the New York hospitals in the United States, the result of the collaboration across private, public, community, and federal hospital systems was able to develop multiple strategies for communication, surge capacity, clinical guidelines, and staff wellness during the COVID‐19 pandemic.^17^ However, Shi et al. from Korea state that while tertiary hospitals are crucial for treating both COVID‐19 patients and patients without the virus, hospital staff must safeguard themselves against unforeseen in‐hospital transmission. For safeguarding tertiary hospitals and their staff during the COVID‐19 epidemic, a comprehensive response must be implemented.^18^ This can also be evident in the reports in Iran as well, demonstrating a high rate of COVID‐19 infection among healthcare workers.^19^, ^20^ Through applying proper hospital policies, better management of healthcare workers can be achieved to avoid high exposure and burnout during these stressful circumstances. However, these policies should be assessed from a broader viewpoint while considering the impact of hospital downgrading and closure on the overall mortality rates and concurring events. These applied implantations, although imperfect, should be evaluated in various settings to provide experience and data for future policymakers. There are few similar studies conducted at the time of the pandemic. In South Korea, a study showed an increase in inpatient mortality any time there was an emergency department closure. These findings are in the same line with those of our study.^21^

Chronic kidney disease patients who were followed and evaluated in India showed a significant increase in morbidity and mortality due to limited or delayed access to dialysis services. In our study, dialysis patients were not separately assessed, but deaths due to diseases of the genitourinary system were shown to have a decreasing trend which might be due to a difference in healthcare systems in Iran and India; for example, there are dialysis units which are not strictly dependent on hospitals in Iran, which provide the chronic kidney disease patients with care, so their sessions are not delayed at the times of hospital downgrading.^22^

There are some limitations in our study. First, since downgrading and hospital closure and corresponding events have been rarely reported in the literature; also, in our country we were unable to compare our implanted policies and outcomes with other similar events, thus making our report merely descriptive for future policymakers. Another limitation is that we were unable to assess the financial burden and other aspects of hospital downgrading and closure, which could all affect the implementation of these measures. Although our study focused on a vital aspect, which was the mortality rates, aside from providing data on the results and burden of the COVID‐19 pandemic and applied measures, we were able to evaluate the frequency and trends in mortality rates in our country.

## 5 CONCLUSION

Hospital closure due to the COVID‐19 pandemic has had a major effect on mortality rates, while also affecting the trend of mortality along with the admission status in Fars province. However, long‐term evaluation after the reopening of hospitals is warranted to evaluate the exact effect of these imposed conditions. Evaluation of the long‐term impact of hospital closure on disease‐related mortalities should be continuously assessed to better determine the depths of the impact of such conditions. On the other hand, due to different outcomes in certain specific fields with other countries, further comparisons of the impact of hospital closure policies in Iran with other countries and also evaluation of the effect of different imposed policies during hospital closure on mortality rates would be beneficial in reaching a better cost–benefit ratio and optimization of such policies.

## AUTHOR CONTRIBUTIONS

**Mohammad Javad Fallahi**: Conceptualization; resources. **Sarvin Seifbehzad**: Data curation; investigation; validation; writing—original draft. **Mehran Fereidooni**: Investigation; methodology; resources. **Amirmohammad Farrokhi**: Data curation; formal analysis. **Keivan Ranjbar**: Supervision; writing—review and editing. **Reza Shahriarirad**: Conceptualization; data curation; formal analysis; investigation; methodology; project administration; supervision; writing—review and editing.

## CONFLICT OF INTEREST STATEMENT

The authors declare no conflict of interest.

## ETHICS STATEMENT

The present study was approved by the medical ethics committee of the academy (Ethical Code: IR.SUMS.REC.1399.091). The permission was obtained from the medical ethics committee of Shiraz University of Medical Sciences. All experimental protocols were approved by the Ethics Committee of Shiraz University of Medical Sciences. Based on the retrospective nature of our study, written informed consent was waived by the Ethics committee of Shiraz University of Medical Sciences, and their information was obtained from their hospital records. Permission to carry out the study and access patient records was sought from the Shiraz University of Medical Science authorities; the study was conducted in compliance with the relevant guidelines and regulations and the Declaration of Helsinki and was also approved by the ethics committee of the university.

## TRANSPARENCY STATEMENT

The lead author Reza Shahriarirad affirms that this manuscript is an honest, accurate, and transparent account of the study being reported; that no important aspects of the study have been omitted; and that any discrepancies from the study as planned (and, if relevant, registered) have been explained.

## Data Availability

SPSS data of the participants can be requested from the authors.
